# Dissociation of Learned Helplessness and Fear Conditioning in Mice: A Mouse Model of Depression

**DOI:** 10.1371/journal.pone.0125892

**Published:** 2015-04-30

**Authors:** Dominic Landgraf, Jaimie Long, Andre Der-Avakian, Margo Streets, David K. Welsh

**Affiliations:** 1 Veterans Affairs San Diego Healthcare System, San Diego, CA, United States of America; 2 Department of Psychiatry & Center for Circadian Biology, University of California San Diego, La Jolla, CA, United States of America; 3 Department of Psychiatry, University of California San Diego, La Jolla, CA, United States of America; 4 Animal Phenotyping Core, University of California San Diego, La Jolla, CA, United States of America; Technion - Israel Institute of Technology, ISRAEL

## Abstract

The state of being helpless is regarded as a central aspect of depression, and therefore the learned helplessness paradigm in rodents is commonly used as an animal model of depression. The term ‘learned helplessness’ refers to a deficit in escaping from an aversive situation after an animal is exposed to uncontrollable stress specifically, with a control/comparison group having been exposed to an equivalent amount of controllable stress. A key feature of learned helplessness is the transferability of helplessness to different situations, a phenomenon called ‘trans-situationality’. However, most studies in mice use learned helplessness protocols in which training and testing occur in the same environment and with the same type of stressor. Consequently, failures to escape may reflect conditioned fear of a particular environment, not a general change of the helpless state of an animal. For mice, there is no established learned helplessness protocol that includes the trans-situationality feature. Here we describe a simple and reliable learned helplessness protocol for mice, in which training and testing are carried out in different environments and with different types of stressors. We show that with our protocol approximately 50% of mice develop learned helplessness that is not attributable to fear conditioning.

## Introduction

### Learned helplessness as a model of depression-like behavior

Stress, especially unavoidable stress, is known to promote the development of depression in humans. In the field of psychiatry research many different animal models of depression, fear, and mania are used to model important aspects of mood disorders [1–3]. Learned helplessness (LH) is a common stress-related animal model of depression-like behavior [4]. Although originally developed for the purpose of investigating helplessness in particular, researchers have since recognized the strong relationship between LH and depression in a more general sense. A crucial point of LH is the inescapability and uncontrollability of the stressor, which leads to a state of “giving up” (helplessness) that is comparable to observations made in patients with depression [5–8]. Classic LH experiments are usually carried out with two “yoked” groups of animals, an escapable stress (ES) group that is able to avoid or escape from a stressor, and an inescapable stress (IS) group that is exposed to the same duration and intensity of stressor as the ES group but is unable to avoid or escape the stressor [9–11]. The stressor, typically in the form of mild electric shocks, is terminated for both groups when the animals in the ES group perform an operant response to escape the stressor. Although both groups receive exactly the same duration and intensity of shocks during the training sessions, only the animals in the IS group develop helplessness and fail to escape during subsequent testing. Thus, it is the uncontrollability of the stressor that triggers a failure to escape, not the shocks *per se* [12]. However, since LH was established as a useful model for depression-like behavior, the ES group is often not included, and animals are exposed to either inescapable shocks or no shocks during training. Usually, not all animals exposed to inescapable shocks become helpless [13, 14]. In the context of depression research, the existence of such stress-resistant animals is often exploited to allow comparison of vulnerable animals exhibiting depression-like behavior to resistant animals that received the same uncontrollable stressor treatment without exhibiting depression-like behavior [14].

### Trans-situationality

LH can be evoked in many different species, including humans, other mammals, and non-mammals [5, 15]. However, commonly used protocols differ depending on the species. Specifically, when using mice, which are increasingly common model organisms due to the ease of genetic manipulations, LH is often defined as simply a failure to escape, without taking into consideration how the failures were evoked. This leads to certain confusions in the literature. In common mouse LH protocols, training and testing of the mice are conducted in the same or a very similar environment, using grid floors to deliver electric foot shocks [13, 16]. Consequently, the mice may develop not only a helpless state, but also contextual fear of the shock environment (i.e., fear conditioning) [17]. Both will result in increased failures to escape, but the underlying reasons for escape failures are quite different [12, 18]. According to the original definition of LH, reduced escape behavior can only be attributed to a general change in a helpless state, and not contextual fear, if the animals are exposed to different stressors (for example tail shocks vs. foot shocks) and different environments during training and testing, a feature called “trans-situationality” [12, 18]. Several rodent studies have demonstrated that IS potentiates freezing to a context previously associated with shock, which can be interpreted as a conditioned fear response to the shock environment [18–20]. However, one study using the same environment for training and testing demonstrated that mice exposed to IS froze less to the shock environment compared to mice from the ES group [11], an effect hypothesized to reflect adaptive vigilance in the ES group.

Besides changes in context-related freezing behavior, other differences between LH protocols using the same or different environments for training and testing were shown. Importantly, using the same or a different environment in training vs. testing results in differences in the recovery time of helpless behavior. Whereas a trans-situational LH protocol leads to a general but transient state of helplessness in rats, protocols using the same environment for training and testing result in long-lasting changes in escape behavior [21], a hallmark of conditioned fear [18]. In addition, behavioral consequences of the two protocols show different dependencies on serotonin signaling. In a trans-situational LH protocol, suppression of dorsal raphé serotonin transmission prevents escape failures [22, 23]. However, when training and testing are carried out in the same environment, the same serotonergic manipulation has no effect, most likely because escape failures due to fear conditioning still occur independent of serotonin signaling [18]. Moreover, lesions of the basolateral amygdala, a brain area involved in the development of conditioned fear, eliminate the long-lasting changes in escape behavior when using the same environment for training and testing, but have no influence on transient changes when different environments are used [17]. These experiments show that the two methods are fundamentally different. Therefore, especially for studies investigating basic features of helplessness, the use of trans-situational LH protocols is necessary to exclude the confound of contextual fear. Here we present a reliable and simple method of trans-situational LH for mice.

## Methods

### Animals

In this study two different mouse lines were used: mPer2^Luciferase^ (PER2::LUC) and C57BL/6J wild-type (WT) mice (Jackson Laboratory, Sacramento, CA, USA). PER2::LUC knockin reporter mice carry the firefly luciferase (Luc) gene in exon 23 of the WT circadian clock gene *Period2* (*Per2*) [24]. In addition, they incorporate an SV40 polyadenylation site to enhance expression levels and were backcrossed to C57BL/6J background [25]. PER2::LUC is a useful real-time reporter of circadian rhythms in various tissues and single cells. PER2::LUC mice develop normally and show no morphological abnormalities. At the end of the behavioral studies, tissues of PER2::LUC mice were collected and used for experiments investigating circadian rhythms for a different study (data not shown). C57BL/6J WT mice were delivered from Jackson Laboratory to our animal facility at the age of 5 weeks and allowed to acclimate for 3 weeks. For all experiments only male mice at the age of 8 weeks were used.

**Fig 1:**
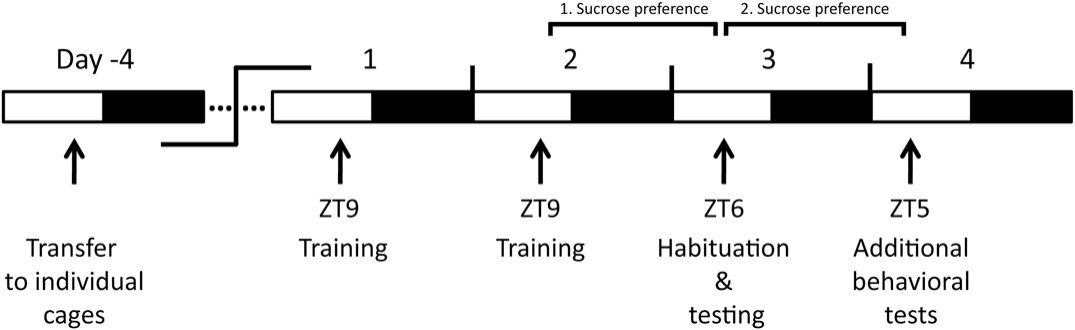
Time course of learned helplessness protocol and subsequent additional behavioral tests. Five days before training, mice were transferred to individual cages. Training on days 1 and 2 was done at ZT9 (3 pm). On day 3 mice were tested three hours earlier at ZT6 (12 noon) in order to prevent time of day related anticipation. At ZT5 (11 am) on the following day, additional behavioral tests (tail suspension test or open field test) were conducted. Acclimation to water bottles used in sucrose testing started 4 days prior to LH testing (for details see “Methods” section). Two sucrose preference tests were conducted for ~1 day each. The first test started immediately after the second training session, and the second test started immediately after the testing session.

Mice were maintained in LD 12:12 cycles (12 h light, 12 h dark, lights on at 06:00 hr) at all times. Five days before the onset of the experiments, mice were separated from each other and single-housed because group housing has been shown to increase vulnerability in the LH paradigm due to altered stress-sensitivity [26]. Mice were randomly assigned to IS and naive control groups and were kept in individually ventilated cages with bedding material and continuous access to water and food. Cages of mice used in the same experiment were kept immediately adjacent to each other on the rack in the housing room. Mice remained in the same cages throughout the whole experiment. After the five acclimation days, mice underwent a three day LH procedure, and some PER2::LUC mice subsequently underwent additional behavioral tests (Fig 1; see below). Each mouse was exposed to only one other behavioral test after LH. Analyses of LH data were done after the subsequent behavioral tests in order to rule out bias in scoring of these other tests. All experiments were carried out in a separate procedure room close to the animal housing room. No animals were excluded from analyses.

Mouse studies were approved by the Institutional Animal Care and Use Committee at University of California, San Diego (Protocol number: S07365). Every effort was made to minimize the number of animals used, and their suffering.

### Learned helplessness

**Fig 2:**
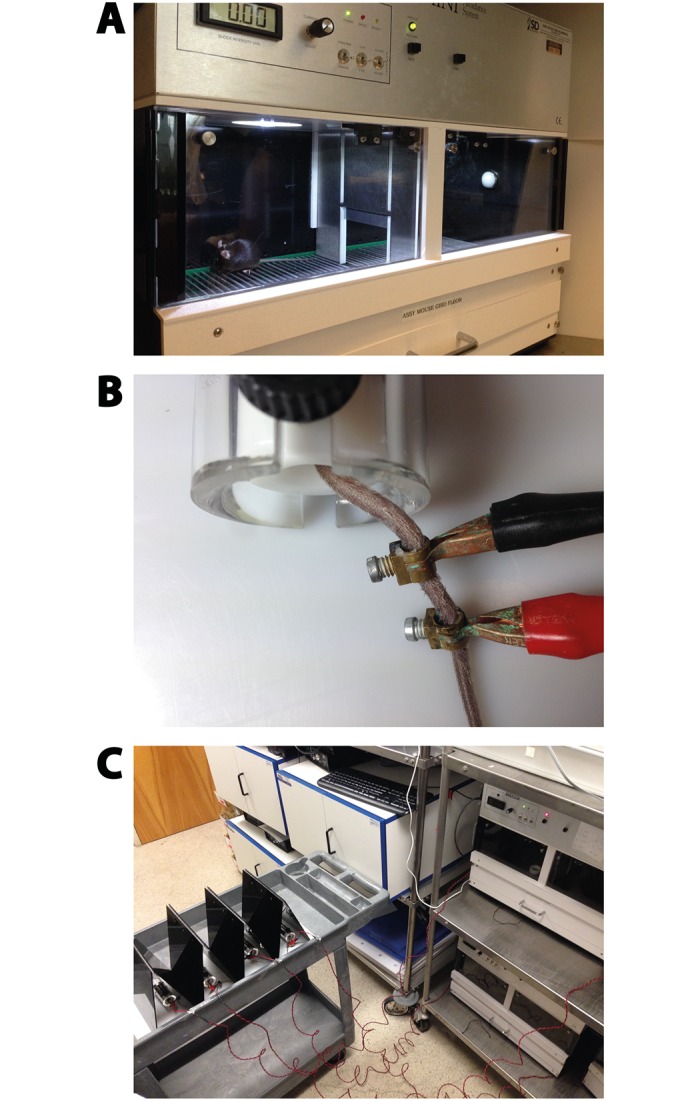
Setup of trans-situational LH. (A) Shuttle boxes are comprised of two compartments separated by a movable gate. Both compartments have a metal grid floor through which the electric foot shocks were delivered. Current intensity can be adjusted for each individual box. Infrared detectors determined the position of the mice at all times during the experiment. (B) During training, mice were kept in a restrainer, and conductive paste was administered to their tails. Conductive metal rings with screws (taken from luster terminals) were gently attached to the tails (about 1 cm apart). Cables that deliver the electric shocks from the shuttle boxes to the mice were connected to the metal rings with alligator clips (red: positive and black: negative). (C) Overview of the whole trans-situational LH setup. Each shuttle box is connected to two cables (positive and negative) so that the electric shocks from the grid floor inside the shuttle box compartments can be delivered to the tails of the mice outside the boxes. Each mouse was connected to one box. The mice were kept in restrainers that were placed on a cart with high borders, which blocked the shuttle boxes from view. In addition, black plastic roofs separated the mice from each other to avoid visual contact. The shuttle boxes were run by a computer (not shown).

LH equipment comprised fully automated shuttle boxes, special extension cables to transmit electric tail shocks outside of the shuttle box, and commercial software (Gemini Avoidance System, San Diego Instruments, San Diego, CA, USA). Shuttle boxes contained two equally sized compartments with electric shock grid floors and infrared sensors that allowed localization of the mice. The boxes were divided by a wall with a closable gate in the middle, which allowed the mice to move between the compartments. All walls of the compartments were black except for the front door, which was clear Plexiglas and served as an observation window during the experiment (Fig 2A). Room lights were used to illuminate the shuttle boxes; no additional lighting was used inside the shuttle boxes. Four boxes were used in parallel, connected to one PC that was operating the shuttle boxes and saving data collected during experiments. Equipment and software were set up before the mice were transferred to the procedure room, in order to allow immediate start of the experiment once the mice arrived in the room. After the experiments, the mice were immediately transferred to their home cages, and the shuttle boxes were thoroughly cleaned with an odor-neutralizing and sterilizing cleaner (Airx 44 ACE, Airex Laboratories, Folcroft, PA, USA).

#### Learned helplessness: different environment for training and testing (“trans-situationality”)

Day 1, *Training*: At *Zeitgeber* time 9 (ZT9 = 9 hours after light onset in housing room = 15:00 hr), singly housed mice were brought to the procedure room in their home cages at the same time and transferred to mouse restrainers (Plas Labs, Lansing, MI, USA). Conductive paste (Weaver and Company, Aurora, CO, USA) was administered to the tails of the mice and small metal rings from luster terminals were gently screwed onto the tails (about 1 cm apart) (Fig 2B). To deliver electric shocks to the tails of the mice, two extension cables (custom made from San Diego Instruments, San Diego, CA, USA) (~200 cm) were individually connected to rods of the grid floor of the shuttle boxes. On the other end, the cables were equipped with alligator clips that were attached to the metal rings on the tails, without causing pain or injuries to the mice (Fig 2B). In order to minimize contextual fear, during the whole procedure the mice were kept at a position in the room were the shuttle boxes were not viewable. In addition, as soon as the cables were attached, mice were separated by plastic partitions to prevent visual contact between them (Fig 2C). Mice were exposed to 120 electric shocks, each of 5 sec duration, randomly given with an intertrial interval (ITI) range of 25 to 35 sec. The shocks started at 0.25 mA and were gradually increased during the course of the training session: after every 15 shocks, the current intensity was increased by 0.05 mA, resulting in a range from 0.25 mA to 0.60 mA. We found that escalating shock intensities during training result in higher numbers of helpless animals. All shocks were delivered within 60 min.

Day 2, *Training*: The conditions were the same as for Day 1.

Day 3, *Testing*: At ZT6 (12:00 hr), mice were brought to the procedure room. On this day, a different route to the procedure room was chosen (compared to the route used during training days) to further minimize contextual fear. Mice were immediately transferred to the shuttle boxes. The gate was open, and the mice had 60 sec acclimation time to explore the box and the open gate. During the habituation time, crossings through the gate were recorded. After the habituation time, PER2::LUC mice received 30 electric shocks each of 0.10 mA amplitude and a maximum duration of 30 sec. WT mice received electric shocks of different intensities (0.10, 0.20, 0.27, or 0.30 mA) and a maximum duration of 30 sec. During the shock, the gate remained open, and mice had a chance to escape the shock by crossing the gate to the adjacent compartment. The schedule in pretrials 1–5 was a fixed ratio (FR) 1 (i.e., crossing the gate once terminated the shock). In the remaining trials 6–30, the schedule was changed to FR-2 (i.e., crossing the gate twice was required to terminate the shock). When FR-1 or FR-2 were accomplished, the shock terminated, and the gate closed for 30 sec until the next trial started. The number of escape failures and the escape latency were used as criteria for helplessness. Data collected during FR-1 pretrials were excluded from analysis, consistent with other LH protocols using an identical escape testing procedure [22]. In order to measure persistence of LH in mice, some PER2::LUC mice were tested 8 days after training. Between training and testing, mice were kept in their home cages and left undisturbed. In order to investigate whether escape failures are based on the pre-exposure to electric shocks during the training sessions, naive mice underwent the same testing procedure as described above. However, these mice were kept in their home cages before they were tested, did not receive any training shocks, and were not restrained.

#### Learned helplessness: same environment for training and testing

Day 1, *Training*: At ZT9 (15:00 hr), mice were brought to the procedure room and immediately transferred to the shuttle boxes. While the gate remained closed, mice were exposed to 120 electric shocks each of 0.15 mA amplitude and 5 sec duration, randomly given with an ITI of 25–35 sec. All shocks were given within 60 min.

Day 2, *Training*: The conditions were the same as for Day 1.

Day 3, *Testing*: The conditions were the same as for *Testing* described above with the exception that the same route to the procedure room was chosen.

### Additional behavioral tests

One day after LH testing at ZT5 (11 am) PER2::LUC mice were brought to the procedure room, and additional tests were conducted to investigate the impact of inescapable shocks on other behaviors, like anxiety, motivation to escape stressful situations, and reward-seeking behavior. Each group of mice was only exposed to one additional behavioral test. After the experiments, the mice were immediately transferred to their home cages, and the equipment was thoroughly cleaned with an odor-neutralizing and sterilizing cleaner (Airx 44 ACE, Airex Laboratories, Folcroft, PA, USA).

#### Open field test

Exploratory locomotor activity in a 5 minute test period was measured in an open field (45 X 45 cm) by an AccuScan apparatus (AccuScan Instruments; Columbus, OH). Total distance, immobility time, time spent in the center, and latency until the center area was entered for the first time were recorded automatically by infrared detectors, and data were transferred to a connected PC.

#### Tail suspension test

Lack of active struggling behavior was measured in the tail suspension test as described previously [27]. Briefly, adhesive tape was used to suspend mice from their tails on a metal bar located 30 cm above a flat surface for 6 min. Plastic tubes were put over the tail to prevent grabbing and climbing up the tail. Immobility was quantified by measuring the amount of time when no whole body movement was observed. Whole body movement was defined as movement of all 4 limbs. Flailing with the front limbs was not counted as movement.

#### Sucrose preference

Starting 4 days before LH, a sucrose preference protocol was initiated based on a previously published protocol [28]. Briefly, for two days mice receive two water bottles (A & B), both filled with normal tap water, in order to accustom the mice to the situation of having two bottles. For the following two days, both bottles were replaced with bottles containing a 1% sucrose solution dissolved in tap water so that mice were acclimated to the sweet taste of sucrose. In order to avoid association of electric shocks with the sweet taste of sucrose, the sucrose bottles were replaced with tap water bottles during the LH procedure. Directly after the training on day 2, bottle A was replaced with a bottle containing 1% sucrose whereas bottle B with tap water remained for two days. To avoid a position bias, bottles A and B were switched on the second day of measurement. Sucrose preference for each mouse was calculated as $100\times \frac{VolA}{\left(VolA+VolB\right)}$.

### Statistical analysis

All statistical analyses were conducted using GraphPad Prism. Details about statistical tests used for individual experiments are indicated in the figure legends.

## Results

### Inescapable shock leads to escape failures in a different environment

**Fig 3:**
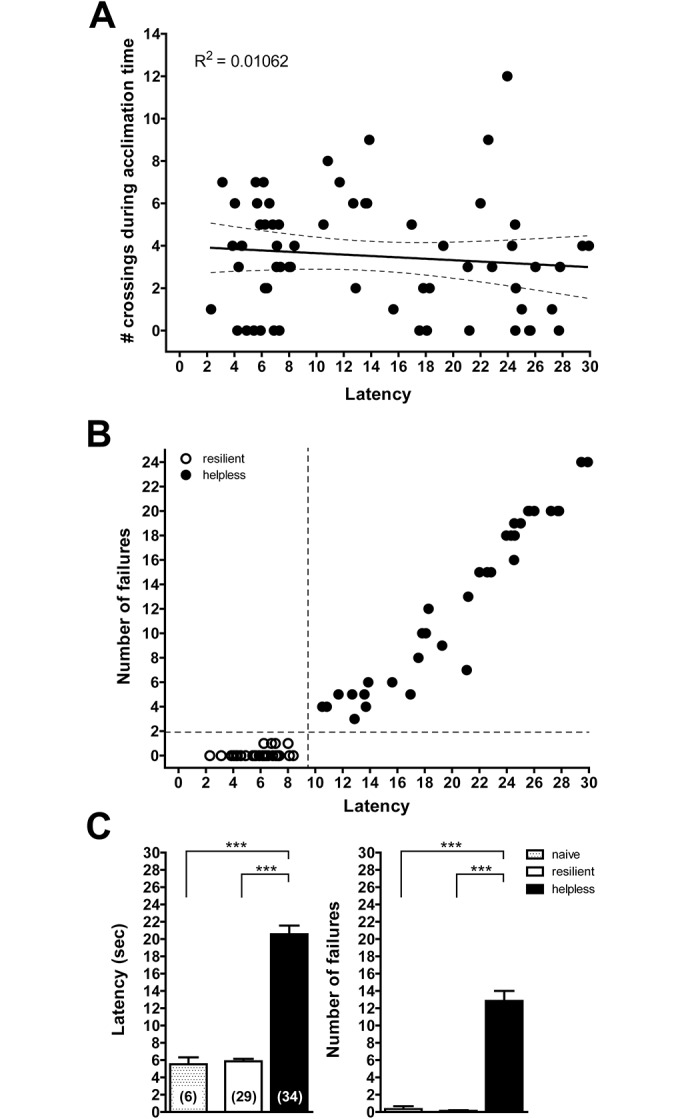
Inescapable electric shocks lead to escape failures in a different environment but not to fear-related behavior in mice. (A) Before testing, mice had a 60 sec acclimation time to explore the shuttle boxes with open gates, and the number of gate crossings was measured. The exploratory behavior of the mice did not correlate with the grade of helplessness subsequently detected. Black line: linear regression line, dashed lines: 95% confidence band, n = 63. (B) After two days of tail-shock training, mice were tested in shuttle boxes, and average escape latency time and number of escape failures were measured. Resilient animals (open circles) were defined as those showing escape latencies within 2 standard deviations of those of naive mice (shorter than 9.5 sec) and numbers of escape failures within 2 standard deviations of those of naive mice (less than 2 failures). Animals with greater escape latencies and larger numbers of escape failures were defined as helpless (black circles). Thresholds are shown as dashed lines. n = 63. (C) Escape latencies and numbers of escape failures of resilient animals were in the range of naive mice that never received training. Data are shown as average ± SEM; ***p ≤ 0.001, (1-way ANOVA with Bonferroni post-test comparing all data sets); n-values are shown in brackets.

After two training days in the restrainer and with tail shocks, PER2::LUC mice were tested for learned helplessness in the shuttle box. Before the FR-1 pretrials began, mice had a 60 sec acclimation time in the shuttle boxes with open gates in order to explore both compartments of the boxes. During that time the number of gate crossings was recorded, which serves as a quantification of exploratory behavior and associatively conditioned fear of the environment. Using the trans-situational LH protocol, mice did not show a correlation between their exploratory behavior during the acclimation in the shuttle boxes and their grade of helplessness (see below) (Fig 3A).

During the five pretrials (FR-1), all mice escaped during at least two trials, demonstrating awareness of the possibility to escape through the gate in order to terminate the shock. During the FR-2 trials, mice exhibited a broad range of numbers of escape failures and latencies (Fig 3B). In order to rule out non-specific escape failures, we also tested naive home cage control mice which never received any training prior to testing. These mice rarely failed to escape and showed very short escape latencies (Fig 3C). Mice that underwent the LH procedure with latencies and failures that fell within 2 standard deviations of those of naive mice, were considered “resilient” to the LH procedure, i.e. resistant to developing learned helplessness (46%). All mice with greater latency and escape failure values were defined as helpless (54%). Thus, training of mice with inescapable tail shocks caused escape failures and long escape latencies in about half of the animals in response to foot shocks applied through the floor of the shuttle box.

### Shock intensity during testing affects escape latency

**Fig 4:**
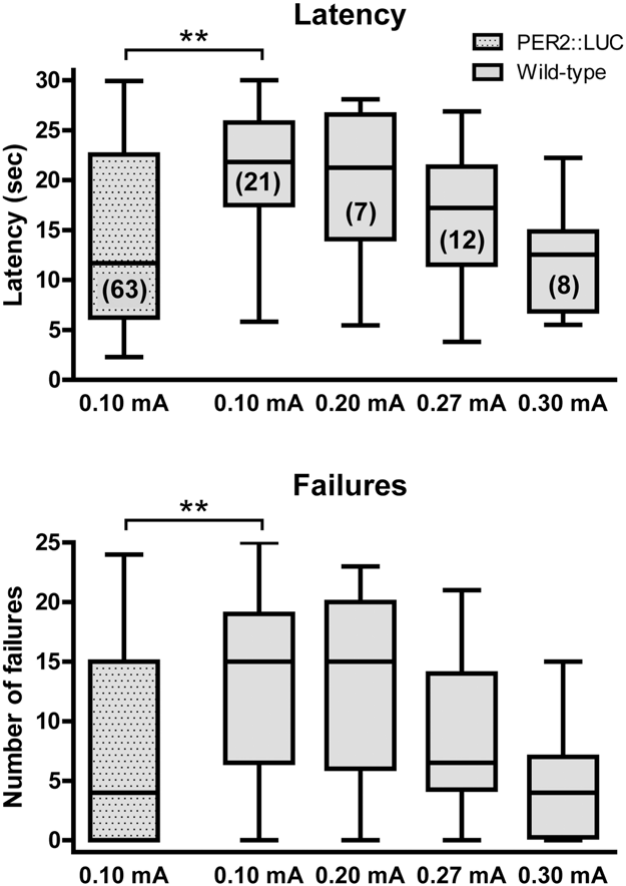
Shock intensity during testing affects escape behavior. Latency times (top panel) and number of escape failures (bottom panel) are shown for PER2::LUC mice tested with 0.10 mA current intensity and WT mice tested with different current intensities ranging from 0.10 mA to 0.30 mA. WT mice required higher current intensities to reach escape latency times and failure frequencies comparable to those of PER2::LUC mice. Data are shown as box-and-whisker plots; **p ≤ 0.01, (1-way ANOVA with Dunnett post test comparing all wild-type data with PER2::LUC data); n-values are shown in brackets.

To test if the trans-situational LH protocol also works in a different mouse line, we exposed C57BL/6J WT mice to the same LH procedure and compared the average escape latency during testing of PER2::LUC and WT mice. Whereas PER2::LUC mice showed an average escape latency time of less than 15 sec and ~5 escape failures when tested at 0.10 mA, WT mice showed a higher average escape latency time of more than 20 sec and more escape failures (Fig 4). However, when the current intensity during testing was increased, WT mice showed a dose-dependent reduction of their average escape latency time and reached values comparable to those of PER2::LUC mice when tested at 0.30 mA (Fig 4B).

### Trans-situational LH is partly correlated with other depression- and anxiety-like behaviors, but not with total locomotor activity

In order to investigate whether trans-situational LH correlates with any other depression- or anxiety-related behaviors, we assessed the effects of inescapable shock on: 1) sucrose preference, a reward-based test; 2) the tail suspension test; and 3) the open field test, an anxiety-based test.

**Fig 5:**
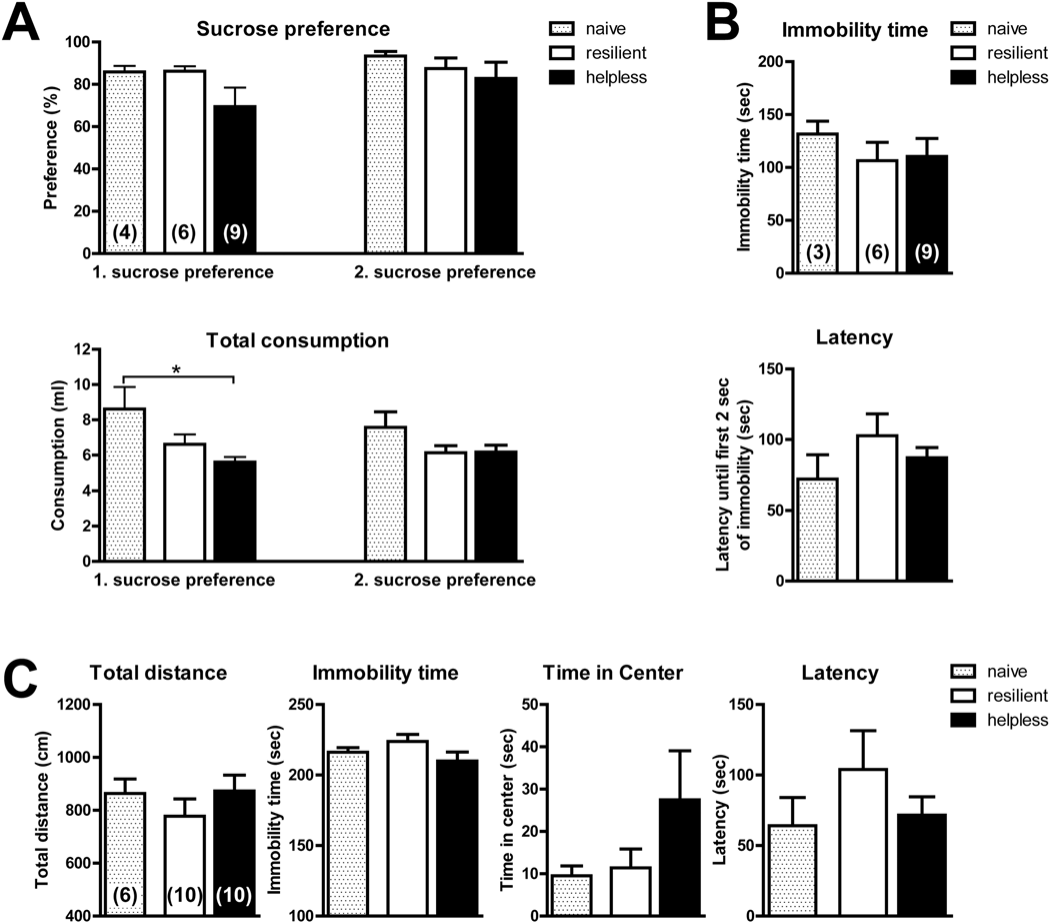
Helplessness is partly correlated with other behavioral phenotypes, but not with total locomotor activity. (A) Sucrose preference test was performed immediately after the second LH training (1. sucrose preference) and a second time after LH testing (2. sucrose preference). Top panel: sucrose preference comparing naive mice that did not receive LH training/testing (spotted bar), resilient (white bars), and helpless mice (black bars). Bottom panel: total consumption of water and 1% sucrose solution of the same mice. Data are shown as average ± SEM; *p ≤ 0.05, (1-way ANOVA with Bonferroni post test comparing all data sets); n-values are shown in brackets. (B) Immobility time (top panel) and latency until the first 2 sec of immobility (bottom panel) in the tail suspension test are not dependent on helplessness. Data are shown as average ± SEM; n-values are shown in brackets. (C) In the open field test, learned helplessness does not change general locomotor activity, on the basis of total distance (left panel) and immobility time (second panel from the left). Helpless mice spend slightly more time in the center of the open area (second panel from the right), but latency until the center is entered for the first time is not different among the groups (right panel). Data are shown as average ± SEM; *p ≤ 0.05, (1-way ANOVA with Bonferroni post test comparing all data sets with each other); n-values are shown in brackets. All tests were carried out in PER2::LUC mice.

Sucrose preference was measured directly after the mice received the second LH training and once again after testing the animals in the shuttle boxes. While the preference for sucrose did not significantly correlate with helplessness, the total liquid consumption of helpless animals was significantly reduced compared to naive home cage controls when measured between the second LH training and testing (1. Sucrose preference) (Fig 5A). However, when sucrose preference was measured again after LH testing (2. Sucrose preference), the total liquid consumption was no longer significantly reduced, suggesting that the LH procedure may acutely reduce liquid intake in mice directly after training (Fig 5A).

The tail suspension test did not reveal any changes in immobility of the animals after trans-situational LH (Fig 5B). Compared to naive home cage controls, mice that underwent the LH procedure did not show significant differences in their total immobility time or in the latency until they first became immobile for two full seconds. Also, whether mice were resilient or helpless did not correlate with either parameter.

Anxiety-related behavior in the open field test did not correlate with helplessness. Helpless mice spent slightly more time in the center of the open field arena than naive home cage controls or resilient mice (Fig 5C). However, this effect was not significant. Importantly, the open field test revealed that mice exposed to the LH paradigm were not impaired in their general locomotor activity since they showed normal levels of total distance traveled in the open field arena and immobility time (Fig 5C).

### Trans-situational LH does not lead to context-related conditioned fear

**Fig 6:**
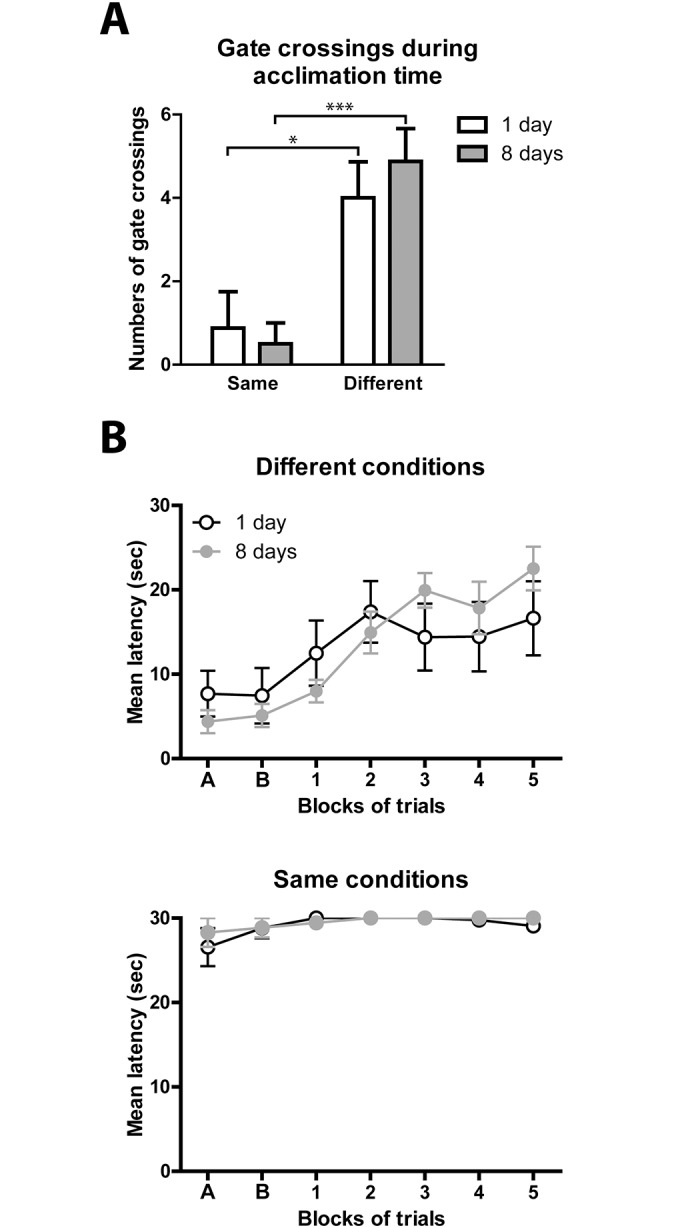
Trans-situationality results in less fear-related behavior in mice. (A) Before mice were tested, they had 60 sec acclimation time to explore the shuttle boxes. The numbers of gate crossings were measured as a marker of contextual fear and exploratory behavior. Data are shown as average ± SEM; *p ≤ 0.05, ***p ≤ 0.001 (Student’s t-test); n = 8. (B) The top panel shows average escape latencies of mice that received training in restrainers with tail shocks, and the bottom panel shows results for mice that received training in one side of the same shuttle boxes where they were subsequently tested. Mice were tested either 1 day after the second training (open circles) or 8 days after the second training (gray circles). A: FR-1 trials 1+2, B: FR-1 trials 3–5, 1–5: blocks of 5 FR-2 trials. Data are shown as average ± SEM; n = 8 (the same mice as in (A); 2-way ANOVAs with Bonferroni post tests did not reveal significant differences between mice tested 1 day or 8 days after training. All tests were carried out in PER2::LUC mice.

We tested the exploratory behavior of all mice during the 60 sec acclimation time before the testing began. Mice that received training and testing inside the shuttle box showed significantly reduced exploratory behavior compared to mice that received IS in the restrainers (Fig 6A). This reduced exploratory behavior was still present when mice were tested 8 days after training. As previously done in rats, we also compared the behavioral consequences of two different LH protocols with training and testing done in different environments or done in the same environment [18]. For both protocols we tested mice either one day or 8 days after the second training session. In contrast to rats, mice did not recover from the LH procedure even after 8 days when training and testing were conducted in different environments, suggesting that mice may be more susceptible to LH than rats and that the effects are longer-lasting than in rats (Fig 6B, top panel). However, mice receiving inescapable shocks in restrainers and those receiving shocks in the shuttle boxes showed remarkable differences. When IS was delivered in a different environment, escape failures mainly occur after several FR-2 trials (Fig 6B, top panel). In contrast, when mice were trained inside the shuttle boxes, they failed to escape during the initial FR-1 trials and almost never attempted to escape during the entire testing procedure (Fig 6B, bottom panel). Consequently, their average latency times are close to the maximum of 30 sec, whereas the group receiving shocks in the restrainers included some animals that always, or almost always, escaped.

## Discussion

### Concept of trans-situationality in learned helplessness can be transferred to mice

Trans-situationality is part of the original definition of LH and was shown to prevent associative conditioned fear in several species [12, 18]. Conditioned fear can be determined by the freezing behavior of animals exposed to the same environment where the stressor was given. Previous studies have shown that freezing behavior increases when the same apparatus was used for training and testing [18–20]. However, using the same environment for training and testing does not invariably lead to increased context-related freezing behavior [11]. Interestingly, amount of freezing behavior is not the only difference between protocols using the same or different environments. In some the species, animals may also show different recovery times and differing involvement of serotonin depending on the protocol. However, to our knowledge, there is no explicit trans-situational LH protocol available for mice. One protocol for mice shows that chronic social defeat potentiates escape failures three days later in a different environment [29]. However, in that protocol, one day before escape behavior to foot shocks was tested, mice received identical shocks in the same testing environment in order to test contextual fear acquisition. Thus, with that protocol, it is difficult to determine whether escape failures were due to helplessness provoked by social defeat or fear-related freezing induced by the prior fear acquisition test. In order to account for the trans-situationality feature of LH in mice, we designed a simple and reliable LH protocol for mice that allows administering an uncontrollable stressor during training in an environment different from the testing environment. In addition, the type of stressor during training and testing differs in our protocol (electric tail shock vs. electric foot shock). To make testing conditions as different as possible from training conditions, a different route to the procedure room was used, and the time of day differed by 3 hours to avoid time-related anticipation. Additionally, mice were placed in a different position in the procedure room and were not able to see the shuttle boxes used for testing. Nevertheless, due to the constraints of our equipment setup, training and testing did occur in the same room, so other cues like odors and sounds could have triggered a fear-related response. However, it is unlikely that fear could account entirely for the helplessness behavior observed in our trans-situational LH procedure. First, approximately half of the mice tested did not develop helplessness and showed no deficits in escape behavior. The generalization of fear from one situation/environment to another would have been expected to produce freezing behavior in most or all mice. Second, in mice that do develop helplessness, escape behavior is intact during the initial testing trials, indicating that a gradual development of escape deficits occurred during the subsequently more demanding FR-2 trials. Third, the presence of a grid floor through which the shocks were delivered was shown to be the most important factor in the development of contextual fear. If shocks are delivered through grid floors, rats freeze as soon as they are placed on another grid floor, even though the apparatus may look different [18]. Thus, we conclude that conditions during training and testing session in our protocol were sufficiently different to avoid fear conditioning. Additionally, since trans-situational LH protocols, which include ES and IS groups, produce helplessness in many other species, including other rodents, we would expect the same to occur in mice, although this needs to be empirically tested.

Using our paradigm and according to our criteria for helplessness, approximately half of the mice became helpless after receiving uncontrollable stress in an environment different from the testing shuttle boxes. The remaining stressed mice were resilient and showed similar behavior to naive home cage control mice that did not receive uncontrollable stress before testing. In line with the trans-situationality concept, our studies in mice showed that, independent of their grade of helplessness, all mice undergoing the trans-situational LH procedure showed similar exploratory activity levels during the testing acclimation time when mice were first introduced to the shuttle boxes. Thus, the trans-situational LH protocol likely does not produce contextual fear in mice, and their behavior reflects a more general helpless state, which applies in different situations and environments [12, 18, 22]. In addition, when comparing mice that received both training and testing in the shuttle boxes with mice undergoing the trans-situational LH protocol, we found that mice that received training shocks in restrainers and had never been in the shuttle boxes before testing only developed escape deficits after multiple testing trials. In contrast, mice trained and tested inside the shuttle boxes showed reduced exploratory activity inside the shuttle boxes on the testing day, presumably due to contextual fear of the environment where they previously experienced inescapable foot shocks. In fact, most mice that were trained and tested inside the shuttle boxes never crossed the gate during the acclimation time. Furthermore, they displayed immediate escape deficits from the first testing trial on. This further suggests increased freezing behavior due to fear of the environment.

In contrast to rats, which recover from trans-situational LH after only a few days [21], helplessness appears to be more persistent in mice. Even when mice were tested 8 days after they received uncontrollable shocks in restrainers, they still showed escape deficits similar to those of mice that were tested one day after training. One explanation might be that mice more readily generalize the fear of electric shocks to different environments compared to rats [30]. LH does not occur in animals that are exposed to ES, but only in animals that receive yoked IS [5]. Indeed, the lack of an ES group is a limitation of the present studies and would help confirm whether mice are generalizing fear of the tail shocks to all electric shocks. A comparison of ES and IS using the present protocol could also clarify whether mice exhibit non-specific escape deficits in the more demanding FR-2 schedule. However, our experiments with naive mice showed that mice are generally capable of escaping from the shocks, even when using the FR-2 schedule. Moreover, while we attempted to make the training and testing environments as different as possible, it should be noted that training and testing were conducted in different locations of the same experimental room. Thus, it is possible that some contextual fear cues contributed to the deficits in escape behavior observed in our studies.

### Differences between mouse lines

Certain mouse lines are more sensitive to LH than others [31]. In our study we compared two different mouse lines, PER2::LUC mice and WT mice, which were both on a C57BL/6J background. Interestingly, PER2::LUC mice require lower current intensities to escape electric shocks in the shuttle boxes than WT mice. One explanation for this result is that PER2::LUC mice may have lower pain sensitivity thresholds than wild-type mice and therefore lower current intensities are sufficient to produce escape behaviors. Alternatively, many mutations of circadian clock genes, including *Per2*, are correlated with depression- or mania-like behavior in mice [32]. Although PER2::LUC mice have a fully functional circadian clock [24], it is possible that other non-circadian functions of the PER2 protein are restricted due to the fusion with the luciferase protein and therefore make these mice more vulnerable to depression-like behavior than WT mice. Nevertheless, our results prove that the trans-situational LH paradigm is effective in both C57BL/6J PER2::LUC and C57BL/6J WT mice. We conclude that different mouse strains show individual sensitivity to mild electric shocks. For that reason, evaluation of the ideal training and testing current intensity might be necessary for different mouse lines.

### Relation between trans-situational LH and other common depression tests

Previous studies have shown that helplessness is correlated with deficits in other behavioral tests [33]. In order to investigate if our trans-situational LH protocol produced further behavioral changes in mice, we performed three additional behavioral tests: sucrose preference, tail suspension test, and open field test.

Although sucrose preference was not correlated to helplessness in mice, we found that total liquid intake was significantly reduced in helpless mice compared to naive home cage controls. This is in line with previous studies showing that, in rats, uncontrollable stress causes a reduction in water intake [34]. In contrast to our results, congenitally helpless rat strains show reduced preference for sucrose [35, 36]. However, these animals were selectively bred for depression-like behavior at baseline whereas in our study helplessness of mice was acutely generated through behavioral training. Furthermore, helplessness may affect reward-related behavior differently in rats and in mice.

The administration of uncontrollable stress leads to reduced swimming in the forced swim test in rats [34]. For our studies, we used the tail suspension test, which is conceptually similar to the forced swim test [1]. The tail suspension test has been proposed to be a measure of despair, but this interpretation is controversial since the test was originally designed as a screening test for antidepressants [37], and the immobility response may occur for reasons other than depression-like behavior [38]. Using our protocol for mice, helplessness did not correlate with immobility time or latency until initial immobility, and mice that underwent the LH procedure showed behavior in the tail suspension test similar to that of naive home cage controls. This suggests that immobility in the tail suspension test is not associated with helplessness behavior, and that behavior in the forced swim and tail suspension tests may respond differently than helplessness to some interventions [1] or may differ between mice and rats.

Importantly, our open field test results showed that general locomotor activity was not affected in helpless mice, since total distance and immobility time were not decreased in helpless mice. Thus, escape failures of helpless mice in our LH paradigm are best accounted for by a change in helpless state, rather than a general deficit in locomotor activity that would prevent the mice from crossing the shuttle box gate. However, anxiety-related behavior in the open field test was not related to helplessness.

## Conclusion

There are two different concepts of learned helplessness: one common LH procedure, predominantly used in mice for drug screens, is to conduct training and testing in the same environment and with the same type of stressor. Most commonly, training shocks are administered in one compartment of the shuttle boxes while the gate remains closed so that the mice have no control over escaping the shocks. The same shuttle boxes are later used for testing, but with an open gate during shocks so that the mice have the possibility to escape. The other LH method is to administer training and testing shocks in different environments, and also to give different types of electric shocks. Both LH procedures result in escape deficits. However, the interpretation of why animals fail to escape is inherently different. When the same environment is used for training and testing, escape deficits are likely the result of conditioned fear rather than helpless behavior. In contrast, when different environments are used for training and testing, escape deficits are likely the result of a general change in helpless state rather than conditioned fear. This consideration is supported by studies showing that the two concepts are neuro-physiologically distinct [18]. Both fear and helplessness are key aspects of depression in humans. Therefore, using protocols with training and testing in the same environment are helpful to investigate fear-related aspects of depression. However, if intended to examine changes in the development of helpless behavior in mice, a trans-situational LH model should be used in order to avoid confusion with fear conditioning.
